# Supplementary material for: The Systems Biology Research Tool: evolvable open-source software
Source: BMC Syst Biol. 2008 Jun 29;2:55. doi: 10.1186/1752-0509-2-55 (PMC2446383; doi:10.1186/1752-0509-2-55)
Supplement: Additional file 1 — SBRT Archive. An archive of the current version of the Systems Biology Research Tool. [file 1752-0509-2-55-S1.zip › sbrt-1.4.0/doc/developers_guide/api/sbrt/ext/R/stat/RCorrelator.html]

RCorrelator


|  |  |  |
| --- | --- | --- |
| **Overview** \| **Package** \| **Class** \| **Use** \| **Tree** \| **Deprecated** \| **Index** \| **Help** | |  |
| PREV CLASS   **NEXT CLASS** | **FRAMES**    **NO FRAMES**     **All Classes** |  |
| SUMMARY: NESTED \| FIELD \| CONSTR \| METHOD | DETAIL: FIELD \| CONSTR \| METHOD |  |


---


## sbrt.ext.R.stat Class RCorrelator<V>

```
java.lang.Object
  sbrt.ext.R.stat.RCorrelator<V>
```

**Type Parameters:**: `V` - the variable type.

**All Implemented Interfaces:**: Correlator<V,RCorrelator.Result>

**Direct Known Subclasses:**: RKendall, RPearson, RSpearman

---

``` public abstract class RCorrelator<V> extends java.lang.Object implements Correlator<V,RCorrelator.Result> ```

This class computes correlation statistics using `R`.

**Author:**
:   This class was written and documented by Jeremiah Wright while in the
    Wagner lab.

---

| **Nested Class Summary** | |
| --- | --- |
| `static class` | `RCorrelator.Result`             This class is used to store the results of computing a correlation using R. |


**Constructor Summary**

`RCorrelator()`


| **Method Summary** | |
| --- | --- |
| `RCorrelator.Result` | `corr(java.util.Collection<java.lang.Double> x, java.util.Collection<java.lang.Double> y)`             Computes and returns the correlation results for the provided values. |
| `RCorrelator.Result` | `corr(java.lang.Double[] x, java.lang.Double[] y)`             Computes and returns the correlation results for the provided values. |
| `RCorrelator.Result` | `corr(java.util.Map<? extends V,java.lang.Double> xValues, java.util.Map<? extends V,java.lang.Double> yValues)`             Computes and returns the correlation statistic of the provided x and y values. |
| `protected abstract  java.lang.String` | `getCorrName()`             Returns the name of the correlation statistic computed by this `R` correlator. |
| `static void` | `main(java.lang.String[] args)`             Used for testing purposes. |

**Methods inherited from class java.lang.Object**

`clone, equals, finalize, getClass, hashCode, notify, notifyAll, toString, wait, wait, wait`

**Constructor Detail**

### RCorrelator

```
public RCorrelator()
```


**Method Detail**

### main

```
public static void main(java.lang.String[] args)
```

:   Used for testing purposes.

    :   **Parameters:**: `args` - ignored.

---


### getCorrName

```
protected abstract java.lang.String getCorrName()
```

:   Returns the name of the correlation statistic computed by this `R`
    correlator. This name should be understood by the `R` function
    `cor.test()`; that is, `kendall`, `spearman`, or
    `pearson` for example.

    :   **Returns:**: the name of the correlation statistic computed by this `R` correlator.

---


### corr

```
public final RCorrelator.Result corr(java.util.Map<? extends V,java.lang.Double> xValues,
                                     java.util.Map<? extends V,java.lang.Double> yValues)
```

:   Computes and returns the correlation statistic of the provided x and y
    values. The key sets of the provided maps need not be identical, but only
    the intersecting values will be considered.

    :   **Specified by:**: `corr` in interface `Correlator<V,RCorrelator.Result>`
    :   **Parameters:**: `xValues` - variables mapped to their values.: `yValues` - variables mapped to their values. **Returns:**: the results of computing the correlation statistic between the provided x and y values.

---


### corr

```
public final RCorrelator.Result corr(java.lang.Double[] x,
                                     java.lang.Double[] y)
```

:   Computes and returns the correlation results for the provided values.

    :   **Specified by:**: `corr` in interface `Correlator<V,RCorrelator.Result>`
    :   **Parameters:**: `x` - an ordered array of values.: `y` - an ordered array of values. **Returns:**: the correlation results for the provided values.

---


### corr

```
public final RCorrelator.Result corr(java.util.Collection<java.lang.Double> x,
                                     java.util.Collection<java.lang.Double> y)
```

:   Computes and returns the correlation results for the provided values.

    :   **Specified by:**: `corr` in interface `Correlator<V,RCorrelator.Result>`
    :   **Parameters:**: `x` - an ordered collection of values.: `y` - an ordered collection of values. **Returns:**: the correlation results for the provided values.


---


|  |  |  |
| --- | --- | --- |
| **Overview** \| **Package** \| **Class** \| **Use** \| **Tree** \| **Deprecated** \| **Index** \| **Help** | |  |
| PREV CLASS   **NEXT CLASS** | **FRAMES**    **NO FRAMES**     **All Classes** |  |
| SUMMARY: NESTED \| FIELD \| CONSTR \| METHOD | DETAIL: FIELD \| CONSTR \| METHOD |  |


---
